# A novel alternative splicing-based prediction model for uteri corpus endometrial carcinoma

**DOI:** 10.18632/aging.101753

**Published:** 2019-01-14

**Authors:** Li Gao, Zu-cheng Xie, Jin-shu Pang, Tian-tian Li, Gang Chen

**Affiliations:** 1 Department of Pathology, First Affiliated Hospital of Guangxi Medical University, Nanning, Guangxi Zhuang Autonomous Region 530021, P. R. China

**Keywords:** alternative splicing, prognostic index, splicing factor, uteri corpus endometrial carcinoma

## Abstract

Alternative splicing (AS) is crucial a mechanism by which the complexity of mammalian and viral proteom increased overwhelmingly. There lacks systematic and comprehensive analysis of the prognostic significance of AS profiling landscape for uteri corpus endometrial carcinoma (UCEC). In this study, univariate and multivariate Cox regression analyses were conducted to identify candidate survival-associated AS events curated from SpliceSeq for the construction of prognostic index (PI) models. A correlation network between splicing factor-related AS events and significant survival-associated AS events were constructed using Cytoscape 3.5. As consequence, 28281 AS events from 8137 genes were detected from 506 UCEC patients, including 2630 survival-associated AS events. Kaplan Meier survival analysis revealed that six of the seven PI models (AD, AP, AT, ME, RI and ALL) exhibited good performance in stratifying the prognosis of low risk and high risk group (P<0.05). Among the six PI models, PI-AT performed best with an area under curves (AUC) value of 0.758 from time-dependent receiver operating characteristic. Correlation network implicated potential regulatory mechanism of AS events in UCEC. PI models based on survival-associated AS events for UCEC in this study showed preferable prognosis-predicting ability and may be promising prognostic indicators for UCEC patients.

Summary: This is the first study to systematically investigate the prognostic value of AS in UCEC. Findings in the presents study supported the clinical potential of AS for UCEC and shed light on the potential AS-associated molecular basis of UCEC.

## Introduction

Endometrial cancer (EC), referred to as uterine corpus endometrial cancer (UCEC), is one of the most common gynaecologic malignancy all over the world and most frequently occurs in postmenopausal women [1,2]. Symptoms arising from UCEC included postmenopausal vaginal bleeding, enlarged uterus, low abdominal pain, and pelvic cramping, which forms the basis of clinical diagnosis [3,4]. There were 569,847 newly registered cases and 311,365 deaths caused by corpus uteri cancer in 2018, causing a serious burden to public health, particularly to people in developing countries [5]. Precision medicine, powered by health record and genetic data of patients, refers to the concept that health care is individually tailored on the basis of a person’s genes, lifestyle and environment. Advances in genomic sequencing has made precision medicine the main melody of current anti-cancer treatment and we attempted to seek reliable genetic changes from the aspect of alternative splicing (AS) to enhance the individualized prognosis prediction of UCEC patients [6].

Alternative splicing (AS) is crucial a mechanism by which the complexity of mammalian and viral proteom increased overwhelmingly [7,8]. Through selective removal of introns and junction of exons, mRNA isoforms with diversified functions can be generated from a single gene [9,10]. AS events occurred in cancer-related genes have significant impact on the progression of human cancers, which is evidenced by the fact that extensive studies reported a large number of AS events in multiple human cancers [11–13]. The occurrence of AS abides by a tissue specific and disease stage specific manner [9].

In UCEC, multiple splice variants of estrogen receptor (ER) and progesterone receptor (PR), two vital molecules that played important roles in the initiation and development of UCEC, were discovered as the results of AS [14]. These receptor variants have been reported to affect the carcinogenesis of UCEC with distinct functions [14–16]. Spliced variants discovered in other genes such as synuclein gamma also implicated the contribution of AS to the tumorigenesis of EC [17]. Convinced of the critical influence of AS events on the tumorigenesis of UCEC, we inferred that AS events might serve as novel prognostic marker for UCEC. Previous studies have indicated that one isoform of ERa: ERaD7 and YT521 exon6-retention mRNA were significantly correlated with the survival of UCEC patients [15,18]. Nevertheless, more unknown AS events in UCEC awaits further excavation. Herein, we pursued the present study on systematically exploring the prognostic significance of AS events in UCEC based on RNA sequencing data in TCGA in order to find promising prognostic predictors for UCEC patients.

## RESULTS

### A preview of survival-associated AS events in UCEC

**Figure 1:**
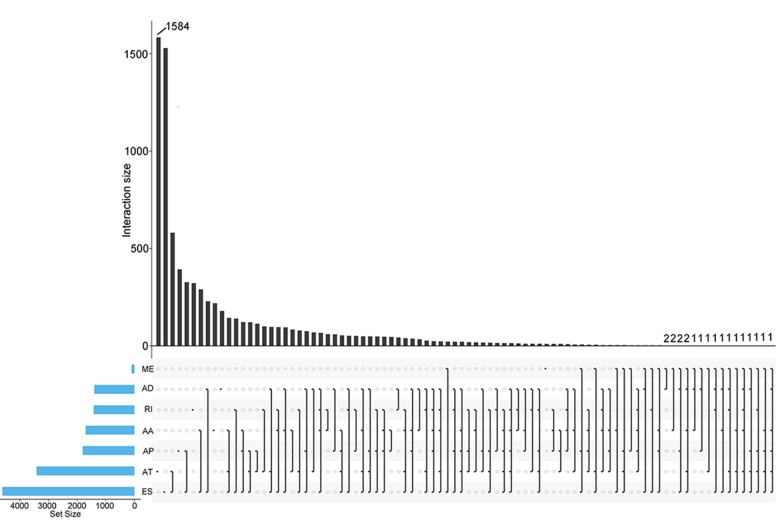
**UpSet plots of AS events in UCEC.** The horizontal axis and vertical axes represent number of genes in the interacting sets of one or multiple AS types and number of gens in each AS type, respectively. AA: alternate acceptor; AD: alternate donor; AP: alternate promoter; AT: alternate terminator; ES: exon skip; ME: mutually exclusive exons; RI: retained intron.

**Figure 2:**
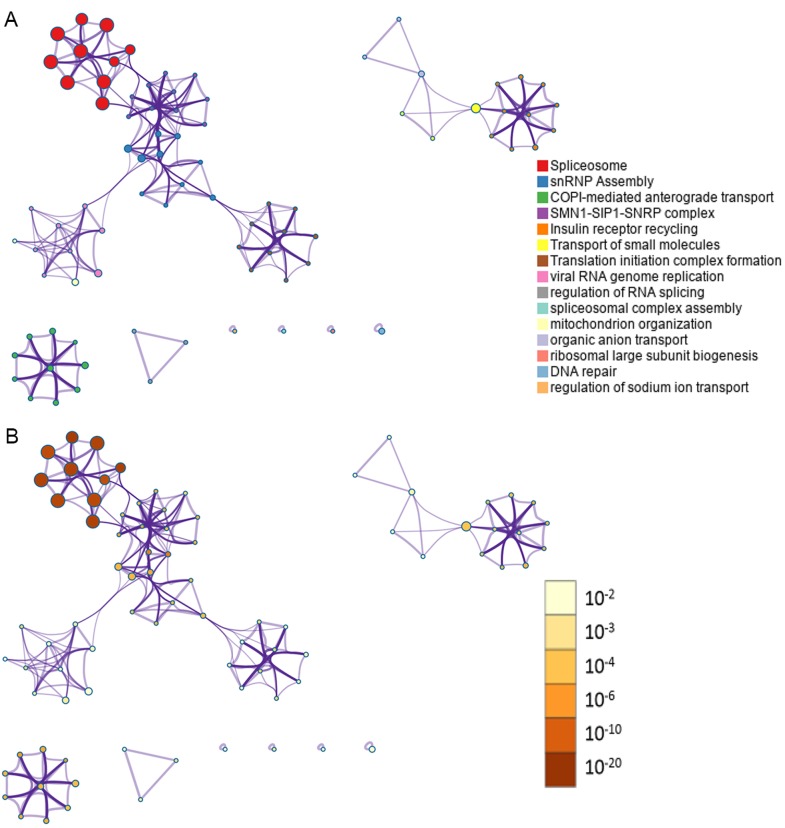
**Network of enriched terms.** (**A**) Nodes in the network represent corresponding genes of top significant survival-associated AS events. One-to-one match between colors of the nodes and enrichment terms were labeled in the left. Nodes that share the same cluster ID are typically close to each other; (**B**) Nodes in the network represent corresponding genes of top significant survival-associated AS events. One-to-one match between colors of the nodes and P values were labeled in the left. Enrichment terms containing more nodes tend to have a more significant P value.

**Figure 3:**
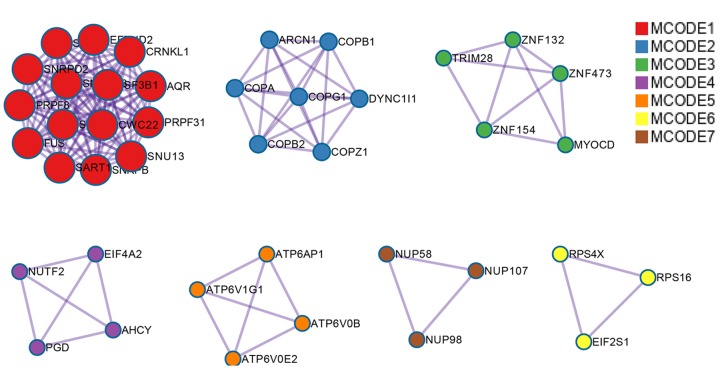
**Protein-protein interaction network for top significant survival-associated AS events.** Nodes in the network represent corresponding genes of top significant survival-associated AS events. Gene that clustered in the same MCODE component was unified in one color.

In total, 28281 AS events from 8137 genes were detected from 506 UCEC patients. Number of AS events identified in seven AS types were recorded in Table 1. For the 28281 AS events from 8137 genes, ES was the predominant type with the maximum number of AS events (n=9744). The intersecting sets of genes and AS events were visualized by UpSet plot in Figure 1, which indicated that one gene might possessed up to six types of AS. With respect to the relationship between AS events and OS of UCEC patients, a total of 2630 survival-associated AS events in 1752 genes were reported from the univariate Cox regression analysis (P<0.05). The distribution of the 2630 survival-associated splicing events in seven AS types was listed in Table 2. We selected top significant survival-associated AS events (P<0.001) to investigate the enrichment of these AS events in biological functions and pathways as well as the interaction network beneath them. The results showed that these significant survival-associated AS events were obviously clustered in biological processes including viral RNA genome replication, regulation of RNA splicing and spliceosomal complex assembly (P<0.01). Top three pathways assembled by these AS events were snRNP Assembly, COPI-mediated anterograde transport and Insulin receptor recycling (P<0.01) (Table 3) (Figure 2). Molecular Complex Detection (MCODE) was used to screen the modules of the protein-to-protein network using the following parameters: degree cut-off = 2, node score cut-off = 0.2, k-core = 2, and maximum depth = 100 [19–21]. Protein-protein interaction network analysis from Metascape for these genes revealed these AS events were gathered in seven MCODE components (Figure 3).

**Table 1:** Summary of AS events in UCEC.

Splicing type	Number of AS events	Number of genes
AA	2270	1691
AD	1877	1386
AP	4458	1792
AT	7796	3411
ES	9744	4604
ME	86	85
RI	2050	1413
Total	28281	10380

Note: AA: alternate acceptor; AD: alternate donor; AP: alternate promoter; AT: alternate terminator; ES: exon skip; ME: mutually exclusive exons; RI: retained intron.

**Table 2:** Survival-associated AS events from univariate Cox regression analysis.

Splicing type	Number of AS events	Number of genes
AA	172	164
AD	170	159
AP	391	227
AT	793	440
ES	929	796
ME	11	11
RI	164	149
Total	2630	1752

Note: AA: alternate acceptor; AD: alternate donor; AP: alternate promoter; AT: alternate terminator; ES: exon skip; ME: mutually exclusive exons; RI: retained intron.

**Table 3:** Pathway and process enrichment analysis for top significant survival-associated AS events.

GO	Category	Description	Count	%	Log10(P)	Log10(q)
CORUM:351	CORUM	spliceosome	17	14.04958678	-18.01041164	-13.69903928
R-HSA-191859	Reactome Gene Sets	snRNP assembly	7	5.785123967	-8.019420114	-4.986801354
R-HSA-6807878	Reactome Gene Sets	COPI-mediated anterograde transport	7	5.785123967	-6.0016261	-3.032676419
CORUM:3118	CORUM	SMN1-SIP1-SNRP complex	3	2.479338843	-5.565637629	-2.652205275
R-HSA-77387	Reactome Gene Sets	insulin receptor recycling	4	3.305785124	-5.020037464	-2.21381508
R-HSA-382551	Reactome Gene Sets	transport of small molecules	13	10.74380165	-3.98898093	-1.462938404
R-HSA-72649	Reactome Gene Sets	translation initiation complex formation	4	3.305785124	-3.622820626	-1.156546304
GO:0039694	GO Biological Processes	viral RNA genome replication	3	2.479338843	-3.484731862	-1.070409087
GO:0043484	GO Biological Processes	regulation of RNA splicing	5	4.132231405	-3.177663215	-0.892370138
GO:0000245	GO Biological Processes	spliceosomal complex assembly	4	3.305785124	-3.010155112	-0.785142581
GO:0007005	GO Biological Processes	mitochondrion organization	9	7.438016529	-2.780755792	-0.606103997
GO:0015711	GO Biological Processes	organic anion transport	8	6.611570248	-2.575952409	-0.422942539
GO:0042273	GO Biological Processes	ribosomal large subunit biogenesis	3	2.479338843	-2.182984218	-0.107140303
GO:0006281	GO Biological Processes	DNA repair	8	6.611570248	-2.070668634	-0.009716275
GO:0002028	GO Biological Processes	regulation of sodium ion transport	3	2.479338843	-2.015633166	0

Note: GO: gene ontology; CORUM: the comprehensive resource of mammalian protein complexes.

### PI models featured by AS events for UCEC

**Figure 4:**
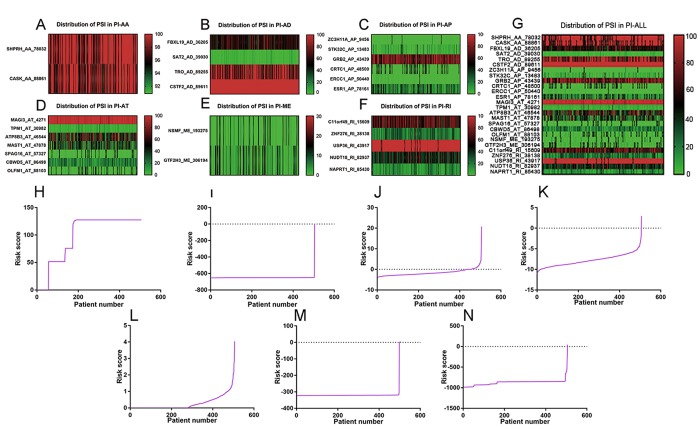
**Distribution of PSI values and risk scores in each PI model.** (**A**) distribution of PSI values in PI-AA model. (**B**) distribution of PSI values in PI-AD model. (**C**) distribution of PSI values in PI-AP model. (**D**) distribution of PSI values in PI-AT model. (**E**) Distribution of PSI values in PI-ME model. (**F**) Distribution of PSI values in PI-RI model. (**G**) Distribution of PSI values in PI-ALL model. The range of PSI values was annotated by a spectrum of colors from green to red; (**H**) Risk scores derived from significant survival-associated AS events in AA type. (**I**) Risk scores derived from significant survival-associated AS events in AD type. (**J**) Risk scores derived from significant survival-associated AS events in AP type. (**K**) Risk scores derived from significant survival-associated AS events in AT type. (**L**) Risk scores derived from significant survival-associated AS events in ME type. (**M**) Risk scores derived from significant survival-associated AS events in RI type. (**N**) Risk scores derived from significant survival-associated AS events in all types.

**Figure 5:**
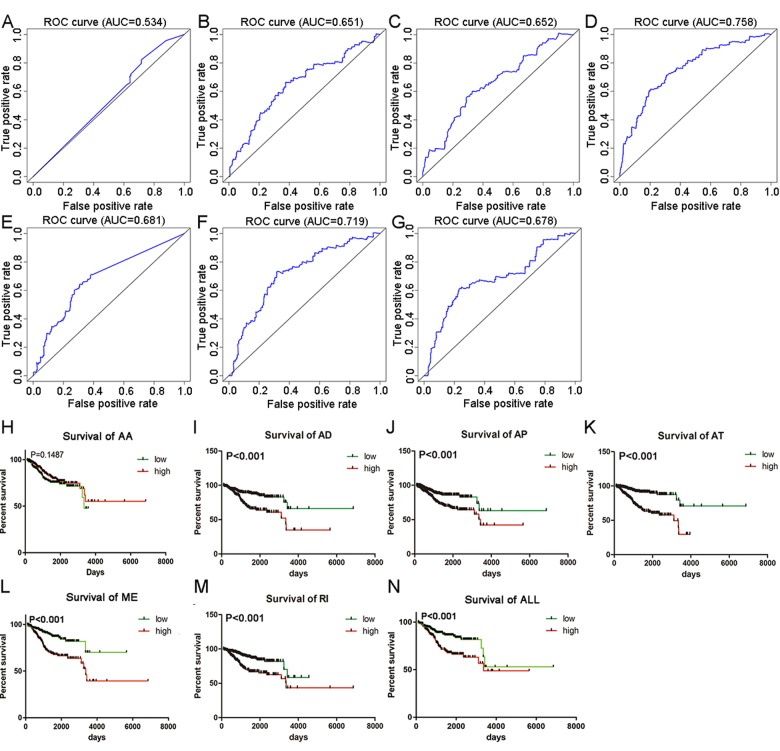
**tROC curves and Kaplan-Meier survival curves seven PI models.** (**A**) tROC curve for PI-AA model (AUC=0.534). (**B**) tROC curve for PI-AD model (AUC=0.651). (**C**) tROC curve for PI-AP model (AUC=0.652). (**D**) tROC curve for PI-AT model (AUC=0.758). (**E**) tROC curve for PI-ME model (AUC=0.681). (**F**) tROC curve for PI-RI model (AUC=0.719). (**G**) tROC curve for PI-ALL model (AUC=0.678). (**H**) Kaplan-Meier survival curves for PI-AA model (P=0.1487). Average OS time in low and high risk group was 2762 and 4713 days, respectively. (**I**) Kaplan-Meier survival curves for PI-AD model (P<0.001). Average OS time in low and high risk group was 5272 and 3219 days, respectively. (**J**) Kaplan-Meier survival curves for PI-AP model (P<0.001). Average OS time in low and high risk group was 5165 and 3469 days, respectively. (**K**) Kaplan-Meier survival curves for PI-AT model (P<0.001). Average OS time in low and high risk group was 5556 and 2511 days, respectively. (**L**) Kaplan-Meier survival curves for PI-ME model (P<0.001). Average OS time in low and high risk group was 4569 and 3848 days, respectively. (**M**) Kaplan-Meier survival curves for PI-RI model (P<0.001). Average OS time in low and high risk group was 3652 and 3957 days, respectively. (**N**) Kaplan-Meier survival curves for PI-ALL model (P<0.001). Average OS time in low and high risk group was 4787 and 3594 days, respectively.

Top significant survival-associated AS events in univariate Cox regression analysis (P<0.001) were considered as candidate AS events for the construction of PI models. Then, multivariate Cox regression analysis was performed to select component AS events for PI models from the 308 top significant survival-associated AS events. According to the results, 26 significant AS events (P<0.05) from multivariate Cox regression analysis were reserved to build PI models of six AS types (AA, AD, AP, AT, ME and RI) (Table 4). Particularly, all the 26 splicing events were merged to construct a PI for all AS types. Distributions of PSI for component AS events and risk scores in each PI model were displayed in Figure 4. The predicting efficiency of the seven PI models was assessed by tROC curves and Kaplan-Meier survival analysis. As illustrated by a panel of tROC curves in Figure 5, PI-AT demonstrated the highest capacity of estimating the prognosis of UCEC patients with an AUC value of 0.758, followed by PI-RI with an AUC value of 0.719. We also used Kaplan-Meier survival analyses to appraise the prognosis-predicting ability of the seven PI models. UCEC patients were separated into low risk and high risk group according to the median values of PI. The results suggested that six of the seven PI models (AD, AP, AT, ME, RI and ALL) exhibited good performance in stratifying the prognosis of low risk and high risk group (Figure 5). Survival time of UCEC patients in low risk group of six PI models (AD, AP, AT, ME, RI and ALL) was significantly prolonged compared to that of UCEC patients in high risk group (P<0.001). According to the assessment from univariate and multivariate Cox regression analysis, four PI models including PI-AP, PI-AT, PI-ME and PI-RI figure prominently with superior independent prognosis-predicting value in both univariate and multivariate Cox regression analysis (Table 5). (all P<0.05). For all PI models, high-risk UCEC patients were more inclined to suffer from advanced clinical progression than low-risk UCEC patients, which is especially obvious for grade classification of UCEC (Table 6).

**Table 4:** Component AS events of PI models for AA, AD, AP, AT, ES, ME and RI.

Splicing type	Gene symbol	AS ID	Exons	From exon	To exon	Hazard ratio	P-value
AA	SHPRH	78032	31.1	30	31.2	0.516	<0.001
AA	CASK	88861	24.1	23	24.2	0.759	<0.001
AD	FBXL19	36205	8.2	8.1	9	1.030	0.004
AD	SAT2	39030	5.2	5.1	6	1.112	0.007
AD	TRO	89255	12.2:12.3	12.1	13	0.986	0.036
AD	CSTF2	89611	12.2	12.1	13	0.001	<0.001
AP	ZC3H11A	9456	4	null	null	1.203	0.041
AP	STK32C	13483	2	null	null	1.072	0.001
AP	GRB2	43439	1	null	null	0.961	0.031
AP	CRTC1	48500	2	null	null	3.785	0.004
AP	ERCC1	50440	1	null	null	1.283	0.001
AP	ESR1	78161	4	null	null	1.048	0.001
AT	MAGI3	4271	23	null	null	0.913	0.018
AT	TPM1	30982	13.2	null	null	1.153	<0.001
AT	ATP8B3	46544	29	null	null	0.978	0.001
AT	MAST1	47878	14.2	null	null	1.015	0.027
AT	SPAG16	57327	14	null	null	1.583	<0.001
AT	CBWD5	86498	17.2	null	null	1.051	0.002
AT	OLFM1	88103	2	null	null	1.090	<0.001
ME	NSMF	193275	6|7	4	9.2	1.075	0.035
ME	GTF2H3	306194	10|11	9	12	1.155	<0.001
RI	C11orf49	15609	14.2	14.1	14.3	1.021	0.045
RI	ZNF276	38138	12.2	12.1	12.3	1.027	0.011
RI	USP36	43917	17.2	17.1	17.3	0.039	0.006
RI	NUDT18	82937	2.2	2.1	2.3	1.019	0.028
RI	NAPRT1	85430	11.5:11.6:11.7	11.4	11.8	1.033	0.008

Note: AA: alternate acceptor; AD: alternate donor; AP: alternate promoter; AT: alternate terminator; ME: mutually exclusive exons; RI: retained intron; P values were calculated from multivariate Cox regression analysis.

**Table 5:** Univariate and Multivariate Cox regression analysis of clinical parameters and seven PI models in TCGA cohort of UCEC patients.

Clinical variable	Group	Univariate		Multivariate	
		HR (95% CI)	P value	HR (95% CI)	P value
Age	<60	2.247 (1.326-3.806)	0.003	1.722 (0.983-3.014)	0.057
	≥60				
Histological type	Endometrioid	2.413 (1.590-3.663)	<0.001	1.112 (0.756-1.635)	0.590
	Serous				
Grade	1-2	1.804 (1.162-2.802)	0.009	1.203 (0.810-1.785)	0.360
	3				
Stage	I-II	3.918 (2.568-5.978)	<0.001	3.527 (2.248-5.533)	<0.001
	III				
PI-AA	Low	0.734 (0.481-1.119)	0.150	0.563 (0.333-0.950)	0.031
	High				
PI-AD	Low	2.446 (1.570-3.813)	<0.001	1.303 (0.781-2.173)	0.310
	High				
PI-AP	Low	2.237 (1.428-.3.503)	<0.001	2.025 (1.270-3.229)	0.003
	High				
PI-AT	Low	4.209 (2.551-6.944)	<0.001	2.102 (1.170-3.776)	0.013
	High				
PI-ME	Low	2.551 (1.620-4.018)	<0.001	2.058 (1.261-3.359)	0.004
	High				
PI-RI	Low	2.560 (1.638-4.001)	<0.001	2.052 (1.283-3.282)	0.003
	High				
PI-ALL	Low	2.057 (1.330-3.181)	0.001	0.983 (0.548-1.763)	0.953
	High				

Note: AA: alternate acceptor; AD: alternate donor; AP: alternate promoter; AT: alternate terminator; ME: mutually exclusive exons; RI: retained intron.

**Table 6:** Relationship between PI models and the progression of UCEC.

Type of PI	Clinico-pathological parameters	Group of risk low % high %		χ^2^	P value
PI-AA	Grade			4.577	0.032
	1-2	113 (27.3)	90 (72.7)		
	3	134 (31.3)	158 (68.7)		
	Stage			0.627	0.429
	I-II	186 (39.1)	178 (60.9)		
	III	67(17.0)	75 (83.0)		
PI-AD	Grade			18.409	<0.001
	1-2	126 (27.3)	77 (72.7)		
	3	124 (31.3)	168 (68.7)		
	Stage			0.979	0.322
	I-II	187 (39.1)	177 (60.9)		
	III	66 (17.0)	76(83.0)		
PI-AP	Grade			1.583	0.208
	1-2	109 (27.3)	94 (72.7)		
	3	140 (31.3)	152 (68.7)		
	Stage			0.352	0.553
	I-II	185 (39.1)	68 (60.9)		
	III	179 (17.0)	74 (83.0)		
PI-AT	Grade			43.455	<0.001
	1-2	139 (27.3)	64 (72.7)		
	3	112 (31.3)	180 (68.7)		
	Stage			20.715	<0.001
	I-II	205 (39.1)	159 (60.9)		
	III	48 (17.0)	94 (83.0)		
PI-ME	Grade			26.660	<0.001
	1-2	132 (27.3)	71 (72.7)		
	3	121 (31.3)	171 (68.7)		
	Stage			1.410	0.235
	I-II	188 (39.1)	176 (60.9)		
	III	65 (17.0)	77 (83.0)		
PI-RI	Grade			25.217	<0.001
	1-2	130 (27.3)	73 (72.7)		
	3	120 (31.3)	172(68.7)		
	Stage			0.352	0.553
	I-II	185 (39.1)	179(60.9)		
	III	68 (17.0)	74 (83.0)		
PI-ALL	Grade			25.974	<0.001
	1-2	130 (27.3)	73 (72.7)		
	3	119 (31.3)	173 (68.7)		
	Stage			6.618	0.010
	I-II	195 (39.1)	169 (60.9)		
	III	58(17.0)	84 (83.0)		

Note: AA: alternate acceptor; AD: alternate donor; AP: alternate promoter; AT: alternate terminator; ME: mutually exclusive exons; RI: retained intron.

### Correlation network of splicing factor-related AS events and survival associated AS events

**Figure 6:**
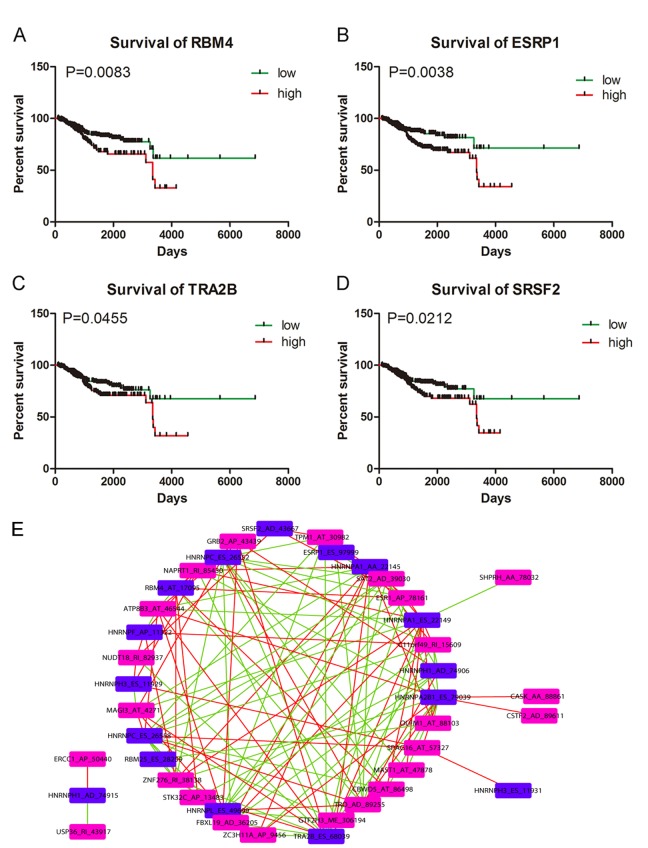
**Kaplan-Meier survival curves for appraising the prognostic significance of splicing factors in UCEC and Correlation network**. (**A**) Kaplan-Meier survival curves for RBM4 (P=0.0083). (**B**) Kaplan-Meier survival curves for ESRP1 (P=0.0038). (**C**) Kaplan-Meier survival curves for TRA2B (P=0.0455). (**D**) Kaplan-Meier survival curves for SRSF2 (P=0.0212). (**E**) Correlation network between splicing factor-related AS events and significant survival-associated AS events. Blue nodes (n=16) and purple nodes (n=24) represented splicing factor-related AS events and significant AS events from multivariate Cox regression analysis, respectively. Positive and negative correlations between splicing events were marked as red lines (n=68) and green lines (n=64), respectively.

We downloaded information of 74 splicing factors and the corresponding splicing factor-related AS events from the SpliceAid2 database and TCGA. Results from univariate Cox regression analysis suggested that 16 splicing factor-related AS events were remarkably linked to the survival of UCEC patients (Supplementary Table 1). The correlation between the 16 splicing factor-related AS events and 26 significant AS events from multivariate Cox regression analysis were calculated and significant correlations were presented as a correlation network in Figure 6E (P<0.05) (Supplementary Table 1). Blue nodes (n=16) and purple nodes (n=24) represented splicing factor-related AS events and significant AS events from multivariate Cox regression analysis, respectively. Positive and negative correlations between splicing events were marked as red lines (n=68) and green lines (n=64), respectively. We also conducted Kaplan-Meier survival analysis for the 12 splicing factors of the 16 splicing factor-related AS events after dividing UCEC patients according to the average expression value of the 12 splicing factors. We found that four splicing factors including RBM4, ESRP1, TRA2B and SRSF2 served as significant prognostic indicators for the worse survival of UCEC patients (P<0.05) (Figure 6A-D).

## DISCUSSION

Existing risk stratification for UCEC patients based on morphological classification had limited power in predicting the overall survival conditions of UCEC patients and it remained unsolved to find effective prognostic indicator for UCEC patients. Accumulated evidence suggested that AS exerted vast influence on the biological events of human cancers [22–24], which enlightened us that the aberrant AS profiles in UCEC may provide valuable prognostic information. Although multiple splice variants of molecules such as ER and PR have been reported in previous studies to participate in the pathogenesis of UCEC, there lacks systematic and comprehensive analysis of the prognostic significance of AS profiling landscape for UCEC.

The present study is the first to investigate global pattern of survival-associated AS events in UCEC using TCGA data. Results from univariate Cox regression analysis revealed that thousands of AS events were associated with the survival of UCEC patients (P<0.05). Subsequent functional annotation for genes corresponding to the significant survival-associated AS events in UCEC (P<0.001) indicated that these genes were mainly involved in biological processes and pathways including viral RNA genome replication, regulation of RNA splicing, spliceosomal complex assembly, snRNP Assembly, COPI-mediated anterograde transport and Insulin receptor recycling. AS events generated from these genes might affect the initiation and development of UCEC through interfere with the above biological processes and pathways.

Bioinformatics analysis of the significant survival-associated AS events in UCEC is one of the highlights of our research. The most striking clinical implication of the present study was that we constructed PI models with noticeable predicting power for overall survival of UCEC patients. Six PI models exhibited significant ability in prognosing overall survival time of UCEC patients with PI-AT performing best (AUC=0.758). More encouragingly, univariate and multivariate Cox regression analysis proved four of the PI models including PI-AP, PI-AT, PI-ME and PI-RI as independent prognostic factors for UCEC. Prior to our studies, several researchers have developed prognostic models for UCEC based on other genomic signatures. Zhou M et al. have devised a lncRNA-focus expression signature for survival prediction in UCEC, which achieved excellent prognostic performance (AUC=0.887) [25]. The high AUC value in the study of Zhou M et al. was derived from a validation cohort of 151 UCEC patients while 506 UCEC patients in our study were designated as validation groups for PI. AUC values of a six-gene signature proposed by Wang Y et al. and a nine-gene prognostic model devised by Ying J et al. reached 0.787 and 0.82, respectively [26,27]. The difference values between these two gene-expression signatures and PIs in our study were within the range of ±0.1, which indicated that the prediction efficiency of PIs in this study were comparable with gene-expression models. In the current study, UCEC cases enrolled in the prognostic analysis were restricted to those whose OS time exceeded 90 days and the validation cohorts were composed of 506 UCEC patients from TCGA, which is different from previous study with similar works. It is understandable that a certain amount of error was inevitable due to the inclusion criteria of patients with prognostic data and heterogeneity of validation cohort. Results in our study offered a novel visual angle for the precision medicine of UCEC patients and the molecular mechanism of tumorigenesis of UCEC. Evaluation results from tROC curves and Kaplan-Meier survival curves proved that building PI models based on survival –associated AS events was a feasible way to stratify UCEC patients into risk groups of different survival outcome.

It is well known that AS may introduce nonsense-mediated mRNA decay, truncated protein and increased or decreased miRNA binding sites, eventually changing the quality and quantity of protein product. Additionally, splicing events in untranslated regions or non-coding RNAs might lead to abnormal gene function [28]. Cancer-specific mRNA transcripts may affect the formation and progression of human cancers via activating oncogenes or inhibiting tumour suppressor genes [29]. Splice-switching of MYO1B into an oncogenic isoform drove gliomagenesis [30]. Presence or absence of exon7 in two splicing isoforms of MBNL1 conveyed opposite phenotypical implications of cancer [31]. Two splicing isoforms of ZNF148 exerted mutual antagonistic effect to each other on the biological activities of colorectal cancer [32]. Throughout the 26 corresponding genes of component AS events for PI models, four genes were closely associated with UCEC. In the study of Wong YF et al., OLFM1 was found to display significant down-regulation in endometrial cancer of Hong Kong Chinese women [33]. Oestrogen receptor α (ESR1) has great impact on the susceptibility and prognosis of endometrial cancer [34]. Latest research discovered that five adjacent tag single-nucleotide polymorphisms at the 5’ end of ESR1 denoted lower risk of UCEC [35]. Significant correlation was established between single nucleotide polymorphisms of ERCC1 and chemosensitivity of UCEC in the study conducted by Chen L et al. [36]. Elevated GRB2 was engaged in oncogenic events of UCEC triggered by insulin [37]. Apart from the four genes, other genes such as FBXL19, CSTF2, ZC3H11A, CRTC1 and MAGI3 influenced the formation and progression of human cancers with either carcinogenic or tumor-suppressive function [38–42]. Although none of the corresponding AS events of the 26 genes was reported in UCEC, it is conjectured that loss-of function for the tumor suppressor gene or gain-of-function and retain-of-function for the oncogene induced by AS may connect the 26 component AS events in PI models to the cancer biology of UCEC.

As critical regulators of splicing events, the prognostic significance of splicing factors and the correlation between splicing factor-related AS events and survival-associated AS events are also worthy of exploration. Correlation network in this study depicted the complicated interactions between splicing factor-related AS events and survival-associated AS events. Both positive and negative correlations were observed between one splicing factor-related AS event and multiple survival-associated AS events; or one survival associated AS event and multiple splicing factor related AS events. For example, SERBP1_AA_3354 was negatively correlated with HNRNPA1_AA_22145 and was positively correlated with HNRNPC_ES_26552. We speculated that splicing factors might execute diversified regulatory functions in mediating AS events of UCEC. Moreover, assessment from Kaplan-Meier survival analysis indicated that four splicing factors including SRSF2, TRA2B, ESPR1 and RBM4 were all associated with the worse survival of UCEC patients. Of note, SRSF2 was linked with poor survival of patients with myelodysplastic syndromes and the frequent mutation of SRSF2 could induce oncogenesis in hematopoietic cells through activating a cascade of alternative splicing [43]. Expression of TRA2B served as independent prognostic factor for the worse progression-free survival of UCEC patients in the study of Ouyang YQ1 et al. [44], which were in concordance with our results. However, the prognostic significance of ESRP1 and RBM4 in this study was conflicting with documents in previous studies. ESRP1 is a kind of epithelial cell-specific epithelial cell-specific alternative splicing controller with involvement in epithelial–mesenchymal transition (EMT) [45,46]. ESRP1 could suppress tumorigenic potential in various cancers including colorectal cancer, pancreatic cancer and ovarian cancer [47–49]. Similarly, RBM4 was reported to inhibit tumor progression via specifically controlling splicing related to the apoptosis, proliferation, and migration of cancer cells [50]. Whether the expression of ESRP1 and RBM4 indicated good or poor clinical outcome of UCEC patients require further investigations in future studies.

Although PI models with impressive predicting power were produced in this study, limitations of the present research should also be pointed out. The prediction efficiency of PIs in this study was not the best among all prognostic models to date. Functional annotation of the genes corresponding to significant survival-associated AS events were theoretical analysis based on public databases. The regulatory network was constructed on the correlations calculated between PSI values of splicing factor-related AS events and survival-associated AS events. Experiments were warranted in future studies to validate the functional role of survival-associated AS events in UCEC and the stimulating or inhibitive influence of splicing factors on AS events.

In conclusion, we identified PI models based on survival-associated AS events for UCEC with preferable prognosis-predicting ability. Findings in this study were anticipated to provide novel options for selecting reliable prognostic indicators for UCEC patients. Furthermore, the correlation network between splicing factor-related AS events and survival-associated AS events may deepen the understanding of the carcinogenesis of UCEC.

## MATERIALS AND METHODS

### Process of AS data curation

TCGA data portal (https://portal.gdc.cancer.gov/) provided the RNA sequencing data of UCEC cohorts. Analysis of mRNA splicing profiles in UCEC was conducted with the aid of SpliceSeq [51], a java program that explicitly quantifies RNA-Seq reads and identifies its possible functional changes as a consequence of AS in the context of transcript splice graphs. We downloaded the percent spliced in (PSI) value for seven types of AS events: Exon Skip (ES), Mutually Exclusive Exons (ME), Retained Intron (RI), Alternate Promoter (AP), Alternate Terminator (AT), Alternate Donor site (AD) and Alternate Acceptor site (AA) to quantify AS events in UCEC. PSI value is a commonly used ratio for the scoring of AS events from zero to one.

### A preview of survival-associated AS events in UCEC

A total of 506 EC patients were included in this study and the overall survival (OS) of the 506 included EC patients were at least 90 days. Since it would be extremely intricate to illustrate the relationship between five or more interactive sets, we used UpSetR (version 1.3.3) [52] rather than Venn diagram to present the intersections between seven types of AS. Interactive sets among the seven types of AS events were visualized by UpSet plot. Based on the median cut of each parameter, these patients were divided into two groups. Univariate Cox regression analysis was conducted to identify survival-associated AS events (P<0.05). Functional enrichment analysis and gene network of top significant survival-associated AS genes (P<0.001) in UCEC were imported from Metascape, (http://metascape.org), an online tool that incorporates resources including KEGG Pathway, GO Biological Processes, Reactome Pathway Database, Canonical Pathways and CORUM to provide functional annotation for genes. Significant terms met the criteria of P value < 0.01 and the number of enriched genes ≥ 3.

### PI models featured by AS events for UCEC

Multivariate Cox regression analysis was applied to top significant survival-associated AS events (P<0.001) selected from univariate Cox regression analysis in each AS type for further evaluation of the prognostic value of AS events in UCEC. AS events with P <0.05 from multivariate Cox regression analysis were retained to construct prognostic index (PI) for the corresponding AS type, which was calculated from the following formula: PI=$\sum_{i}^{n}PSIi*\beta i$ (β means the regression coefficient). To compare the efficiency of PI models for each AS type, survival ROC package (version 1.0.3) in R (version 3.3.0) that enables time-dependent receiver operating curves (tROC) estimation to accommodate censored data [53] was employed to calculate area under the curve (AUC) value for the tROC curves of each PI model. Kaplan-Meier survival curves were also used to compare the prognostic ability of prediction models. P values reported from all analyses were two-sided. To examine the independence between PI models and important clinical features, we performed univariate and multivariate Cox regression analysis to compare the hazard ratio (HRs) of PI models and important clinical features for UCEC. Furthermore, the relationship between PI models and clinical progression of UCEC was calculated through Chi square test in SPSS v.22.0.

### Correlation network of splicing factor-related AS events and survival associated AS events

Splicing factors played indispensable role in regulating splicing events [54]. In this study, we dived deeper into the underlying molecular mechanism of AS events in UCEC through exploring the correlation network of splicing factor-related AS events and survival associated AS events. We obtained the information of splicing factors from SpliceAid2 (www.introni.it/spliceaid.html) and downloaded the level 3 mRNA-seq expression data of the splicing factors from TCGA data portal. Considering the rationality of transcripts per million (TPM) format in the interpretation of RNA-seq data [55], primitive count values were converted into TPM. We conducted univariate Cox regression analysis to assess the association between OS of UCEC patients and PSI of splicing factor-related AS events in TCGA. Whether significant correlation existed between PSI of survival-associated splicing factor-related AS events (P<0.05) and distinct AS events from multivariate Cox regression analysis (P<0.05) were judged by Spearman correlation test. Interactions between survival-associated splicing factor-related AS events and distinct AS events from multivariate Cox regression analysis were displayed in the form of correlation network by Cytoscape (version 3.5.0). Adjusted P values were considered significant when less than 0.05.

### Statistical analysis

Statistical analysis for this study has been detailed in previous study [56].

## Supplementary Material

Supplementary Table
