# Supplementary material for: A novel alternative splicing-based prediction model for uteri corpus endometrial carcinoma
Source: Aging (Albany NY). 2019 Jan 14;11(1):263–83. doi: 10.18632/aging.101753 (PMC6339785; doi:10.18632/aging.101753)
Supplement: Supplementary Table [file aging-11-101753-s001.docx]

**SUPPLEMENTARY MATERIAL**

**Supplementary Table 1. Pearson Correlation analysis between splicing factor-related AS events and top survival-associated AS events.**

| Splicing factor-related AS event | Top significant survival-assocaited AS events | P value | r |
| --- | --- | --- | --- |
| HNRNPA1_AA_22145 | FBXL19_AD_36205 | 0.009699289 | -0.120872411 |
|  | SAT2_AD_39030 | 2.63E-12 | -0.304650253 |
|  | TRO_AD_89255 | 6.10E-06 | 0.201511246 |
|  | ZC3H11A_AP_9456 | 0.049513772 | -0.088421716 |
|  | ESR1_AP_78161 | 1.07E-05 | -0.212774966 |
|  | MAGI3_AT_4271 | 0.013368917 | 0.109911733 |
|  | TPM1_AT_30982 | 2.38E-07 | -0.227234781 |
|  | ATP8B3_AT_46544 | 0.003216382 | 0.138449647 |
|  | OLFM1_AT_88103 | 0.003583272 | -0.137038472 |
|  | GTF2H3_ME_306194 | 6.16E-04 | -0.162808767 |
|  | C11orf49_RI_15609 | 0.004428813 | -0.129017431 |
|  | ZNF276_RI_38138 | 7.78E-08 | -0.237404071 |
|  | NAPRT1_RI_85430 | 0.012015834 | -0.112589268 |
| SRSF2_AD_43667 | GRB2_AP_43439 | 0.0001 | 0.319 |
|  | ESR1_AP_78161 | 0.005 | -0.137 |
|  | TPM1_AT_30982 | 0.0001 | -0.173 |
|  | NUDT18_RI_82937 | 0.031 | -0.103 |
| HNRNPH1_AD_74906  www.aging-us.com 1 AGING  www.aging-us.com 1 AGING | FBXL19_AD_36205 | 0.0001 | 0.234 |
|  | SAT2_AD_39030 | 0.001 | 0.15 |
|  | TRO_AD_89255 | 0.0001 | -0.165 |
|  | ZC3H11A_AP_9456 | 0.003 | 0.132 |
|  | STK32C_AP_13483 | 0.002 | 0.139 |
|  | GRB2_AP_43439 | 0.001 | -0.151 |
|  | C11orf49_RI_15609 | 0.007 | 0.121 |
| HNRNPH1_AD_74915 | ERCC1_AP_50440 | 0.014 | -0.118 |
|  | USP36_RI_43917 | 0.001 | 0.156 |
| HNRNPF_AP_11322 | ATP8B3_AT_46544 | 0.0001 | 0.168 |
|  | C11orf49_RI_15609 | 0.0001 | -0.17 |
|  | ZNF276_RI_38138 | 0.01 | -0.117 |
|  | NUDT18_RI_82937 | 0.022 | -0.109 |
|  | NAPRT1_RI_85430 | 0.031 | -0.098 |
| RBM4_AT_17095 | TRO_AD_89255 | 0.045 | 0.09 |
|  | STK32C_AP_13483 | 0.044 | -0.09 |
|  | GRB2_AP_43439 | 0.038 | -0.097 |
|  | ESR1_AP_78161 | 0.043 | -0.098 |
|  | ATP8B3_AT_46544 | 0.011 | 0.119 |
|  | CBWD5_AT_86498 | 0.0001 | -0.197 |
|  | ZNF276_RI_38138 | 0.011 | -0.114 |
|  | NAPRT1_RI_85430 | 0.009 | -0.116 |
| HNRNPH3_ES_11929 | SPAG16_AT_57327 | 0.0001 | -0.228 |
|  | NUDT18_RI_82937 | 0.048 | -0.093 |
|  | NAPRT1_RI_85430 | 0.028 | -0.099 |
| ESRP1_ES_97999 | TRO_AD_89255 | 0.0001 | 0.194 |
|  | ESR1_AP_78161 | 0.02 | 0.121 |
|  | TPM1_AT_30982 | 0.0001 | -0.291 |
| HNRNPC_ES_26558 | FBXL19_AD_36205 | 0.048 | 0.093 |
|  | SAT2_AD_39030 | 0.006 | 0.123 |
|  | TRO_AD_89255 | 0.0001 | -0.169 |
|  | STK32C_AP_13483 | 0.021 | 0.104 |
|  | ESR1_AP_78161 | 0.009 | 0.128 |
|  | MAGI3_AT_4271 | 0.019 | -0.105 |
|  | TPM1_AT_30982 | 0.0001 | 0.16 |
|  | ATP8B3_AT_46544 | 0.002 | -0.145 |
|  | SPAG16_AT_57327 | 0.049 | -0.091 |
|  | CBWD5_AT_86498 | 0.02 | 0.103 |
|  | GTF2H3_ME_306194 | 0.007 | 0.129 |
|  | C11orf49_RI_15609 | 0.015 | 0.11 |
|  | ZNF276_RI_38138 | 0.001 | 0.154 |
| HNRNPA1_ES_22149 | SHPRH_AA_78032 | 0.007 | 0.133 |
|  | FBXL19_AD_36205 | 0.041 | -0.096 |
|  | SAT2_AD_39030 | 0.0001 | -0.439 |
|  | TRO_AD_89255 | 0.004 | 0.13 |
|  | ESR1_AP_78161 | 0.0001 | -0.214 |
|  | MAGI3_AT_4271 | 0.011 | 0.114 |
|  | ATP8B3_AT_46544 | 0.0001 | 0.235 |
|  | MAST1_AT_47878 | 0.002 | -0.142 |
|  | CBWD5_AT_86498 | 0.0001 | -0.189 |
|  | OLFM1_AT_88103 | 0.0001 | -0.179 |
|  | GTF2H3_ME_306194 | 0.0001 | -0.174 |
|  | C11orf49_RI_15609 | 0.0001 | -0.219 |
|  | ZNF276_RI_38138 | 0.0001 | -0.387 |
|  | NAPRT1_RI_85430 | 0.0001 | -0.303 |
| HNRNPC_ES_26552 | FBXL19_AD_36205 | 0.0001 | 0.169 |
|  | SAT2_AD_39030 | 0.0001 | 0.355 |
|  | TRO_AD_89255 | 0.0001 | -0.172 |
|  | GRB2_AP_43439 | 0.001 | -0.151 |
|  | ESR1_AP_78161 | 0.0001 | 0.194 |
|  | MAGI3_AT_4271 | 0.001 | -0.154 |
|  | TPM1_AT_30982 | 0.0001 | 0.221 |
|  | ATP8B3_AT_46544 | 0.0001 | -0.238 |
|  | MAST1_AT_47878 | 0.033 | 0.101 |
|  | CBWD5_AT_86498 | 0.0001 | 0.16 |
|  | GTF2H3_ME_306194 | 0.007 | 0.13 |
|  | C11orf49_RI_15609 | 0.0001 | 0.319 |
|  | ZNF276_RI_38138 | 0.0001 | 0.256 |
|  | NAPRT1_RI_85430 | 0.011 | 0.114 |
| RBM25_ES_28259 | SAT2_AD_39030 | 0.003 | 0.151 |
|  | OLFM1_AT_88103 | 0.023 | 0.124 |
|  | ZNF276_RI_38138 | 0.037 | 0.108 |
| HNRNPA2B1_ES_79039 | CASK_AA_88861 | 0.021 | -0.119 |
|  | SAT2_AD_39030 | 0.001 | 0.151 |
|  | TRO_AD_89255 | 0.0001 | -0.192 |
|  | CSTF2_AD_89611 | 0.0001 | -0.204 |
|  | ZC3H11A_AP_9456 | 0.022 | -0.103 |
|  | ATP8B3_AT_46544 | 0.0001 | -0.179 |
|  | MAST1_AT_47878 | 0.025 | 0.106 |
|  | SPAG16_AT_57327 | 0.005 | -0.129 |
|  | CBWD5_AT_86498 | 0.023 | 0.101 |
|  | OLFM1_AT_88103 | 0.003 | 0.139 |
|  | GTF2H3_ME_306194 | 0.038 | 0.099 |
|  | C11orf49_RI_15609 | 0.002 | 0.144 |
|  | ZNF276_RI_38138 | 0.0001 | 0.209 |
|  | NAPRT1_RI_85430 | 0.001 | 0.144 |
| TRA2B_ES_68039 | FBXL19_AD_36205 | 0.033 | 0.1 |
|  | SAT2_AD_39030 | 0.0001 | 0.327 |
|  | TRO_AD_89255 | 0.0001 | -0.159 |
|  | ZC3H11A_AP_9456 | 0.0001 | 0.276 |
|  | STK32C_AP_13483 | 0.013 | -0.112 |
|  | GRB2_AP_43439 | 0.0001 | -0.245 |
|  | ESR1_AP_78161 | 0.0001 | 0.198 |
|  | MAGI3_AT_4271 | 0.001 | -0.155 |
|  | TPM1_AT_30982 | 0.0001 | 0.254 |
|  | ATP8B3_AT_46544 | 0.0001 | -0.19 |
|  | GTF2H3_ME_306194 | 0.0001 | 0.202 |
|  | C11orf49_RI_15609 | 0.0001 | 0.344 |
|  | ZNF276_RI_38138 | 0.0001 | 0.283 |
|  | NUDT18_RI_82937 | 0.003 | 0.138 |
|  | NAPRT1_RI_85430 | 0.004 | 0.129 |
| HNRNPH3_ES_11931 | SPAG16_AT_57327 | 0.015 | -0.113 |
| HNRNPL_ES_49699 | FBXL19_AD_36205 | 0.01 | 0.12 |
|  | SAT2_AD_39030 | 0.0001 | 0.352 |
|  | TRO_AD_89255 | 0.0001 | -0.235 |
|  | STK32C_AP_13483 | 0.019 | 0.105 |
|  | GRB2_AP_43439 | 0.0001 | -0.175 |
|  | ESR1_AP_78161 | 0.017 | 0.117 |
|  | MAGI3_AT_4271 | 0.0001 | -0.194 |
|  | TPM1_AT_30982 | 0.003 | 0.131 |
|  | ATP8B3_AT_46544 | 0.003 | -0.139 |
|  | GTF2H3_ME_306194 | 0.0001 | 0.217 |
|  | C11orf49_RI_15609 | 0.0001 | 0.238 |
|  | ZNF276_RI_38138 | 0.001 | 0.152 |
